# Patients Taking Direct Oral Anticoagulants (DOAC) Undergoing Oral Surgery: A Review of the Literature and a Proposal of a Peri-Operative Management Protocol

**DOI:** 10.3390/healthcare8030281

**Published:** 2020-08-20

**Authors:** Saturnino Marco Lupi, Arianna Rodriguez y Baena

**Affiliations:** 1 Department of Clinical Surgical, Pediatric and Diagnostic Sciences, University of Pavia, 27100 Pavia, Italy; 2 Department of Dentistry, IRCCS San Raffaele Hospital, 20123 Milan, Italy; arianna_rodriguez@hotmail.it; 3 Dental School, Vita Salute University, 20132 Milan, Italy

**Keywords:** DOACs, coagulation, surgery, peri-operative planning

## Abstract

Patients on anticoagulant therapy for the prevention of cardiovascular accidents present an increased risk of bleeding following dental and oral surgery. Four recently introduced non-vitamin K antagonist oral anticoagulants, namely dabigatran etexilate (direct thrombin inhibitor), rivaroxaban, apixaban, and edoxaban (Xa factor direct inhibitor), are widely spreading for convenience of use compared to the older drug class. Dental management of patients taking these drugs has substantial differences compared to patients on vitamin K antagonist therapy. Anticoagulation is not assessed directly through a hematological test, but indirectly by renal function. The interventions must be scheduled at the time of minimum blood concentration of the drug. Bleeding can occur even after several days following the surgery. The interaction with drugs administered for dental care must be carefully evaluated. The peri-operative diet can influence the risk of bleeding. Local measures favoring coagulation must be adopted. The interventions with higher risk must be divided into multiple less invasive interventions. Although antidotes exist for these drugs, their use does not seem necessary for dental interventions that have been planned optimally. Furthermore, in this review of the literature a decision protocol is proposed for the evaluation of the suspension of the anticoagulant drug before oral surgery. Cessation of any anticoagulant should only be made in consultation with the patient’s general practitioner/cardiologist, who will weigh up the risk of bleeding from the proposed procedure with the risk of thrombosis/stroke in each individual patient.

## 1. Introduction

In the last fifty years vitamin K antagonists (VKA), like warfarin, have been considered the oral anticoagulant of choice. The narrow therapeutic index of this drug poses some problems such as recurring appointments to check the coagulation status, frequent dose adjustments, and multiple food and drugs interactions [1].

The need to overcome these issues led to the development of direct oral anticoagulants (DOACs). By a selective and specific action on the individual components of the coagulation cascade, these new molecules provide a more predictable anticoagulating effect.

In UK in 2015, DOACs accounted for more than half of oral anticoagulant prescriptions, with rivaroxaban prescribed most frequently, followed by apixaban and then dabigatran, with an increasing trend [2].

DOACs have enormous advantages: they guarantee a more predictable response; they do not require a constant monitoring of the patient; they are administered at fixed doses, facilitating adherence to therapy; they show minimal drugs and food interactions; and they have a wide therapeutic margin. However, DOACs also have some disadvantages, such as double daily administration in some cases [3].

The poor knowledge of the peri-operative management of patients who take DOACs can determine a great variability in the management of patients undergoing oral surgery [4]. Still in 2019, a survey showed that among practitioners there is a need for empirical-based practical guidelines for the management of patients taking DOACs undergoing oral surgery [5].

The lack of consensus amongst physicians on DOAC discontinuation may reflect conflicting best practice advice. Peri-procedural cessation may also represent a source of confusion for patients [6,7]. On the other hand, VKAs create a significantly greater burden for the patient during peri-procedural management for dental extraction compared with DOACs [8].

By the revision of the parameters that affect the peri-operative management of the patient being treated with DOACs, the purpose of this review was to provide clinical indications and to propose a protocol for the management of the patient ongoing oral surgery.

## 2. Methods

A comprehensive literature search covering the period August 2005—May 2020 was conducted using International Pharmaceutical Abstracts, PubMed, and Ovid MEDLINE to locate review articles, guidelines, and clinical trials that were appropriate and relevant for this review. The following terms were used in the literature search: Pradaxa, Xarelto, Eliquis, Savaysa, dabigatran, rivaroxaban, apixaban, edoxaban, atrial fibrillation (also AFib), venous thromboembolism (also VTE), factor Xa inhibitors, direct thrombin inhibitor, direct oral anticoagulant (also DOAC), new oral anticoagulant (also NAO).

### 2.1. What are DOACs?

The acronym DOAC stands for direct oral anticoagulant, and includes a group of medications composed of direct thrombin inhibitors (DTI) and inhibitors of factor Xa (FXaIs). When these drugs were introduced it was possible to refer to them also with the acronym NAO, which stands for new oral anticoagulant. Since ’new’ could only be used correctly for a limited period of time, the acronym NAO has fallen into disuse and now only DOAC is recognized as valid.

Dabigatran etexilate (Pradaxa^®^) is the prodrug of dabigatran (originally known as BIBR 953), a DTI. Being highly polarized, dabigatran is not widely available orally; therefore, the prodrug of dabigatran, dabigatran etexilate, was developed to facilitate gastrointestinal absorption [9]. After oral administration, dabigatran etexilate is rapidly hydrolyzed by non-specific, ubiquitous esterases to the active form, dabigatran [10]. Rivaroxaban (Xarelto^®^), apixaban (Eliquis^®^), and edoxaban (Savaysa^®^) are orally administered, selective, reversible FXaIs [10,11].

### 2.2. How do DOACs Work?

Dabigatran etexilate is a potent, non-peptidic small molecule that specifically and reversibly inhibits both free and clot-bound thrombin by binding to the active site of the thrombin molecule so that it cannot catalyze fibrinogen into fibrin [9,12,13,14].

Rivaroxaban, apixaban, and edoxaban bind directly to the factor Xa (FXa), which is a protease that converts prothrombin (factor II) to thrombin, used to convert fibrinogen to fibrin and resulting in clot formation. As a result, when compared to DTI, FXaIs act on the previous phase of the coagulation cascade, so that no thrombin is present [15].

The selection of a FXa inhibitor is important, as the factor serves as a medium between the intrinsic and extrinsic coagulation pathways. FXa inhibitors demonstrate a dual mechanism of action by inhibiting free FXa and the FXa produced by prothrombinase, leading to an overall reduction of thrombin. Rivaroxaban and apixaban reversibly inhibit FXa bound within the prothrombinase complex as well as the free enzyme, while edoxaban inactivates only the clot-bound FXa [10,11].

### 2.3. What are DOACs Used For?

DOACs are used for prevention and treatment of cardiovascular events, embolism, and deep venous thrombosis (DVT). Specific indications are listed below in Table 1 [11,15,16,17,18,19,20,21,22,23,24]. Knowledge regarding DOAC indications might be useful when taking patients medical history, in order to identify medical conditions not reported by the patients.

### 2.4. Half-Life, Daily Administration, and Posology

In order to safely perform oral surgery procedures, the knowledge of DOACs’ half-life and dosage is crucially important. Pharmacological properties of the DOACs are listed below in Table 2 [9,10,11,12,13,14,15,20,21,22,23,25,26,27].

Dabigatran can be administered once or twice a day, apixaban is administered twice a day, and rivaroxaban and edoxaban once a day. Time to maximum plasma concentration is between 1 and 4 h. For the mean plasma half-life of each drug, see the Renal function Section 2.9 and Table 4.

### 2.5. Which Tests are Used for Measuring Anticoagulation?

Although DOACs influence on the most common coagulation tests (activated partial thromboplastin time (aPTT), prothrombin time (PT), international normalized ratio (INR)) vary markedly, they are less relevant due to the high variable response linked both to instrumental and physiological conditions. Therefore, they are considered less useful for evaluating the patient’s coagulation. On the contrary, more precise laboratory tests, such as thrombin clotting time (TT), ecarin clotting time (ECT), dilute prothrombin time, and FXa inhibition assay are not commonly performed. Moreover, a factor that greatly influences the coagulation test is the time elapsed between the blood sampling and the last DOAC intake [12,28]. In fact, the maximum effect on the clotting assay corresponds to the peak of plasma concentration, that varies depending on the drug and on the physiological conditions. Therefore, in case of procedures with a low risk of bleeding, the coagulation status in patients using DOAC can be indirectly estimated knowing the drug pharmacological parameters and the patient’s health conditions.

Despite the fact that Mauprivez and Coll. [29] have found a significant increase in aPTT in cases of bleeding in patients undergoing oral surgery, such test is not considered very indicative.

TT and ECT were the most sensitive clotting assays in patients taking dabigatran [9,12]. While measurements of aPTT may provide a qualitative indication of dabigatran anticoagulant activity, like other DTIs [30], it is not suitable for the precise quantification of anticoagulant effect especially at high plasma concentrations of dabigatran. Dabigatran has little effect on the PT at clinically relevant plasma concentrations. Both the ECT and the TT, which are particularly sensitive to the effects of DTI, display a linear dose-response with therapeutic concentrations of dabigatran [25].

Rivaroxaban prolongs PT and aPTT, with the PT being more sensitive than the aPTT depending on the reagents used for testing. However, the effect of the drug on these tests is short-lived, with prolongation only seen at peak drug levels. FXa inhibition is the best test to monitor drug concentrations in plasma [10,27].

Apixaban prolongs the INR and the aPTT in a concentration-dependent fashion. However, its effect on these tests is minimal at therapeutic concentrations and these tests are not predictive of oral surgery bleeding. It can be monitored using a factor Xa inhibition assay or a dilute prothrombin time [10].

Edoxaban has dose-dependent effects on aPTT, PT, and INR values and anti-FXa activity. INR returns to baseline levels within 12 h; prolonged increases in aPTT and PT values occur, but those values return to baseline levels within 24 h after edoxaban dosing. Anti-factor Xa activity also increases in a dose-dependent manner with edoxaban use. Bleeding time is independent of dosing, formulation, or diet factors [11].

The evaluation of coagulation with laboratory tests can be considered unnecessary before dental extractions except for specific patients with renal or liver dysfunction [31].

### 2.6. Drug Interactions

The uptake, metabolism, and elimination of DOACs is influenced by the P-glycoprotein transponder, which is strongly involved in the re-secretion of the drug after absorption in the intestine, and FXaIs are subject to hepatic clearance by CYP3A4-type cytochrome P450-dependent [11,15,25,32]. Moreover, the P-gp transporter may also be involved in renal clearance [33]. As a result, medications metabolized by these enzymes may affect the coagulation status of the patient. P-glycoprotein inhibitors, like verapamil, dronedarone, amiodarone, and quinidine, increase DOAC plasma levels, while inducers, like rifampicin and carbamazepine, reduce plasma drug concentration [33,34]. Strong P-glycoprotein inducers that can be used in dentistry include rifampicin and dexamethasone. Strong P-gp inhibitors include ketoconazole, and moderate P-gp inhibitors include clarithromycin and itraconazole. Strong inhibitors of both CYP3A4 and P-gp include clarithromycin, erythromycin, and systemic azole-antimycotics, such as ketoconazole, itraconazole, voriconazole, and posaconazole. Clinical effects of clarithromycin and erythromycin are not considered to be clinically relevant. Because non-COX-selective non-steroidal anti-inflammatory drugs (NSAIDs; and salicylates) inhibit platelet aggregation and may cause gastrointestinal bleeding and peptic ulceration and/or perforation, it may be prudent to increase monitoring of the patient for signs and symptoms of bleeding if these drugs are used concomitantly, especially in the context of oral surgery procedures. Moreover, clinical trials have shown increased bleeding with concomitant use of DOACs and NSAIDs, as well as rivaroxaban and opioids [25,32,33,34,35,36].

Interestingly, in some studies, which reported delayed bleeding up to six days after surgery, a non-COX-selective NSAID was administered to control pain and other studies did not mention the pain control therapy prescribed [20,37,38]. Paracetamol (acetaminophen) can be used and has proven to be adequate for controlling post-operative pain [3,4,36,39].

Interactions between drugs used in dentistry and oral surgery and DOACs are listed in Table 3.

### 2.7. Antidots

While until a few years ago the absence of effective antidotes represented the major flaw of DOACs, recently some antidotes for reversal of DOACs, like idarucizumab, andexanet alfa, and ciraparantag, have been introduced [40]. There are limited data relating to their use. In addition, they are intended for the management of serious bleeding that can compromise life. In the clinical management of patients undergoing dental care and oral surgery, the use of these antidotes may be excessive since bleeding can be managed in other ways (see the Improvement of Coagulation (Section 2.18)).

### 2.8. Elimination Pathway

All the DOACs are cleared by the kidneys to a different degree (Table 2). The elimination pathway for dabigatran is predominantly renal, while for the other drugs the renal pathway accounts for less than 50%. Renal function alterations can cause drug accumulation and consequently affect coagulation. Renal excretion of unchanged dabigatran is the predominant elimination pathway, with about 80% of an intravenous dose excreted unchanged in the urine. The remainder is conjugated with glucuronic acid to form acylglucuronides, which is predominantly excreted via the bile with only very small amounts of conjugates found in urine. These conjugates are pharmacologically active [11]. Approximately 66% of the rivaroxaban dose is excreted via the kidneys, and the remainder is excreted in the feces [10].

Approximately one-third of apixaban and edoxaban are excreted by the kidneys, whereas the remainder appears in the feces [10,11].

### 2.9. Renal Function

When planning dental procedures in patients treated with DOACs, the evaluation of renal function is very important. Good renal function demonstrates an acceptable anticoagulation state. On the other hand, it allows for achieving coagulation recovery by discontinuing the medication if hemorrhagic events occur.

Although the renal elimination pathway is crucial in the case of DOACs, they are considered a reasonable choice for anticoagulant therapy in atrial fibrillation patients with mild or moderate chronic kidney failure [10,28]. Therefore, the dentist or oral surgeon should not rule out a decrease in renal function in patients taking DOACs.

DOACs half-life trend is shown as a function of creatinine clearance in Table 4 [10]. In the case of patients with impaired renal function, the opinion of the attending physician should always be sought.

In a recent study, a quarter of the bleeds were observed in patients with controlled renal failure. Therefore kidney failure should be considered a bleeding risk factor [39].

### 2.10. Hepatic Impairment

The pharmacokinetic profile of dabigatran is not substantially affected by moderate hepatic impairment (Child-Pugh B) [26].

Rivaroxaban is metabolized in the liver via CYP 3A4, CYP 2J2, and CYP-independent mechanisms. The drug is contraindicated in patients with severe liver disease because metabolic inactivation may be impaired [24,25].

In a recent study [39], a quarter of bleeding was observed in patients with liver disease receiving rivaroxaban. Therefore, liver disease in patients receiving rivaroxaban should be considered a risk factor for bleeding.

### 2.11. Elderly Patients

The increased risk of bleeding for patients over the age of 65 is inferred from the assumption that they are suffering from renal failure [41]. Therefore, the creatinine clearance evaluation in elderly patients is recommended in order to exclude reduced excretory capacity.

In healthy volunteers, dabigatran exposure is 40% to 60% higher in older than in younger participants, reflecting an age-related reduction in creatinine clearance (C_L_C_R_) [25]. On the other hand, clinical studies [3,39] have not found a relationship between the risk of bleeding and old-age in patients who did not have a reduction in kidney function.

### 2.12. Local Risk Factors

Oral surgery is reported to carry ‘no clinically important bleeding risk’ and/or allow adequate local hemostasis’ [28]. This conception conflicts with emergency hospitalizations and with the need for blood transfusions following dental procedures reported in the literature [42].

The extent of the surgical wound is related to the risk of post-operative bleeding. Generally, operations involving the extraction of four or more dental elements and the positioning of three or more endosseous implants are considered high-risk. Therefore, the extraction of up to three teeth and the placement of up to two implants are considered low-risk interventions.

It has recently been highlighted that the extraction of multi-rooted teeth exposes to a greater risk of bleeding [39] and therefore in the case of multi-rooted teeth, the number of teeth that can be extracted in one session must be reduced.

### 2.13. Continuing or Discontinuing the Treatment?

Discontinuation or continuation of DOAC treatment depends on many factors, such as cardiovascular or venous event risk as a result of discontinuation, renal function, and bleeding risk associated with surgery.

For procedures where local hemostasis is possible and/or carrying no important bleeding risk, European Heart Rhythm Association (EHRA) guidelines consider it safe to perform elective surgical intervention at trough level (i.e., ≥12 or 24 h after last intake), suggesting that it may be more practical to have the intervention scheduled 18–24 h after the last intake, and then restart 6 h later (i.e., with skipping one dose for BID NOAC) [28].

Therefore, it seems important to define which interventions can be defined as having no important bleeding risk Table 5.

The extraction of a maximum of three dental elements caused bleeding in 3–17.8% of the procedures [6,29,31,36,37], without a significant increase in the risk of serious bleeding [3,7,31,36,43]. However, cases of serious bleeding are documented even in the case of up to three tooth extractions, although the authors agree with the non-necessity to suspend DOAC in most cases [7,29]. Moreover, a recent study has found that even when three contiguous teeth are extracted, bleeding may occur when these are multi-rooted teeth [39]. When continuing anticoagulant therapy, an increased risk of bleeding has been reported for more than three simultaneous tooth extractions. Abayon and Coll [44] reported a clinically insignificant bleeding occurred after one day in a patient taking rivaroxaban and subjected to nine concurrent teeth extractions. Breik and Coll reported a severe post-operative bleeding in a patient taking dabigatran and subjected to 18 concurrent teeth extraction [45]. Although it has been reported that DOAC intake does not increase the risk of post-operative bleeding in patients undergoing multiple implant surgery [35], it seems appropriate to limit the number of implants for each surgical session and possibly to divide the placement of implants into sites not contiguous in multiple surgical sessions.

Positioning up to two implants in the posterior region and three implants in the anterior one carries a low risk of bleeding. Gomez-Moreno et al. [46] did not observe an increased bleeding risk in dabigatran-treated patients who underwent up to two dental implants placement in the posterior region and three dental implants in the anterior region. In their clinical trial, patients (age < 75 years) showed no renal function alterations and the flap design was without the releasing incision. According to their study protocol the procedure was carried out 12 h after the last administration of dabigatran and the following dosage was postponed by 8 h after the surgery. The next day the medication was taken regularly. Following surgery, local hemostatic measures were taken consisting of non-absorbable sutures and compression with sterile gauzes soaked in 5% tranexamic acid. Gomez-Moreno et al. [47] did not observe an increased bleeding risk in patients treated with rivaroxaban without modification of the anticoagulant therapy who underwent up to two dental implants placement in the posterior region and three dental implants in the anterior region. In their clinical trial patients (age < 75 years) showed no renal function alterations and the flap design was without the releasing incision. However, the time window between the last administration of the medication and the surgery is not specified in this study. In the case of complex oral surgery (extraction ≥ 4 dental elements) suspension of the DOACs must take into consideration the anticoagulant used, the risk of bleeding and the renal function (Table 6).

It does not appear that it would be necessary to discontinue the use of dabigatran or rivaroxaban before dental treatment, including most uncomplicated tooth extractions, in most patients, especially if adjunctive local hemostatic measures (e.g., absorbable gelatin or oxidized cellulose sponges, sutures, local pressure (with sterile gauze pads moistened with water, normal saline solution, or 5% tranexamic acid solution), etc.) are used appropriately when indicated. However, in situations where oral/maxillofacial surgical procedures may require the temporary discontinuation of dabigatran or rivaroxaban, owing to concerns for possible complications resulting from excessive bleeding and/or impaired hemostasis, dabigatran or rivaroxaban should be discontinued at least 24 h before elective surgery, or longer, depending on the risk of bleeding based on the type and complexity of the surgical procedure, the presence and degree of any renal impairment, and the presence of other risks for impaired hemostasis [15].

Periodontal surgery is considered to be low-risk for bleeding, unless it involves an extension of the flap in the free gingiva [28,48], since the extension of the flap in the free gum involves the invasion of a tissue with multiple blood vessels and easily mobilized.

In the event of placement of a number greater than or equal to four implants, in a recent study in patients receiving rivaroxaban, the DOAC was suspended 24 h before surgery. In this study, only three minor bleeding events treated with compression were observed out of 12 treated patients and 57 placed implants [49].

Continued mono or dual anticoagulation therapy with rivaroxaban (and aspirin), increases post-operative bleeding risk for oral surgical procedures, although the bleeding complications are manageable [20]. Although anticoagulation therapy in general increases the post-operative bleeding risk, and considering that discontinuation of anticoagulation therapy may result fatal (thromboembolic) events [50], continuing anticoagulation therapy, including with rivaroxaban, during oral surgical procedures may be recommended. Furthermore, it is advisable to monitor the patient closely for up to one week with a 24 h hotline available as well as a pre-operative consultation with the primary physician or the cardiologist [20].

Moreover, the discontinuation of anticoagulation therapy with substitution of low-molecular- weight or unfractionated heparin as a bridging regimen also increases the incidence of myocardial infarction, stroke and systemic embolism, hospitalization, and/or death within 30 days. Furthermore, heparin bridging increases the bleeding incidence to 5% [3].

In the literature there are no specific data on the risk associated with the short-term discontinuation of DOACs. Instead, there are data related to the long-term discontinuation of DOACs: among those who discontinued DOAC therapy, the most common reasons were physician preference, patient refusal, high bleeding risk, and other indications. DOAC permanent discontinuations were associated with increased rates of all-cause mortality (8.5 vs. 2.9 events # per 100 patient-years), all-cause hospitalization (64.1 vs. 37.0 events # per 100 patient-years), and major cardiovascular/neurologic adverse events (30.9 vs. 21.8 events # per 100 patient-years) [51].

It should be noted that frequently the cases of bleeding associated with DOAC appeared some days after the operation and the risk of immediately post-operative bleeding is similar to that of patients taking warfarin [31,36,37].

To reduce the risk of long-term bleeding it would be necessary to suspend DOACs for long periods (5–10 days).

Discontinuation of anticoagulant therapy leads to an increased risk of thromboembolic accidents [50,52,53,54]. Therefore, it remains questionable whether it is reasonable to suspend DOACs for relatively long periods of one or two weeks. Moreover, the drug withdrawal instructions may be misunderstood by the patient, with incorrect, prolonged, and unnecessary withdrawal intervals [6,7,55].

Delayed bleeding was not reported in a recent study of 119 patients receiving DOACs. In this study, dental extractions were performed at least 6 h after taking a DOAC and local hemostasis measures, such as suture and collagen sponge, were adopted. In addition, patients were hospitalized for 24 h after extraction and in case of post-operative bleeding, the administration of a DOAC was avoided until hemostasis was achieved [31].

Previously published data regarding post-operative bleeding in patients receiving DOACs undergoing oral surgery are presented in Table 7.

### 2.14. Reintroduction of the Discontinued Medication

If discontinued, administration of DOACs should be restarted after a stable fibrin clot is formed. An advantage of DOACs over warfarin is the rapid achievement of the anticoagulative state without the need to recalibrate the dose after reintroduction.

A post-operative discontinuation from a minimum of 6 to a maximum of 48 h has been adopted depending on the type of surgery [3,4,15]. In a study on implant surgery, dabigatran was suspended 24 h before surgery and was reintroduced 8 h after surgery [46]. In a clinical study on 12 patients undergoing up to three dental extractions, the medication was reintroduced the day after the surgery [3].

### 2.15. Peri-Operative Diet

Although food consumption during the post-operative period may delay the plasma concentration peak of the medication [25], it does not seem to have clinical relevance. In fact, the maximum concentration or the plasmatic concentration over time do not change [12]. In addition, by carrying out the intervention as far as possible from the last intake, the plasma concentration is not affected. The anticoagulant activity of edoxaban is not affected by food intake [57]. On the contrary, fluid intake reduction could lead to drug accumulation and consequent increase of anticoagulation [12]. Therefore, since the patient after surgery could be led to reduce fluid intake, it is important that it is maintained and monitored.

### 2.16. Surgical Planning

Although a correct surgical planning is necessary in all patients [9], those who may require DOACs need to have even more scrupulous planning.

Bleeding in patients taking DOACs can occur not only in the immediate post-operative period, but affects the entire healing period. Delayed bleeding has been observed for up to 13 days after tooth extraction [4]. Therefore, it seems appropriate to plan the surgical interventions at the beginning of the week and away from the periods of holidays, in order to manage the possible appearance of bleeding.

Some authors suggest performing the intervention in the late afternoon, in the case of patients taking DOACs once daily, in correspondence with a decrease in the plasma concentration of the drug [7]. However, it must be considered that carrying out the operation in the morning offers the patient the possibility of finding the surgeon in the hours immediately following the operation.

Performing surgery too short after taking NOAC increases the risk of bleeding. Mauprivez and Coll [29] found a significant increase in bleeding risk in patients undergoing surgery within 4 h from last DOAC dose.

In a recent study, dental extractions were performed at least 4 h after the last DOAC intake. Out of a total of 100 treated patients, four bleeding episodes were observed: three minor bleedings managed by the patient by applying a dressing saturated with tranexamic acid on the post-alveolar socket for 20 min, and one moderate bleeding treated with necrotic clot removal and placement of a new suture. All the bleeding appeared in multi-rooted extraction [39]. Since the bleeding rate was low, but not zero, by performing extractions at least 4 h after taking the DOAC, it seems correct to assume that the longer intervals recommended by the European Heart Rhythm Association (EHRA) may guarantee a lower risk of bleeding. In case of intervention at low-risk of bleeding (e.g., extraction up to three dental elements), EHRA experts suggest not to interrupt therapy with DOACs performing the surgery at trough plasmatic level (i.e., ≥12 or 24 h after last intake) [28]. This protocol has been found effective in preventing post-operative bleeding in a clinical study on teeth extraction, with no serious bleeding observed [3].

In case of extraction of contiguous multi-rooted teeth or extraction of more than three teeth, oral surgeons can split surgery in more than one session [3]. Since it is possible to postpone these surgical procedures in most cases, it is advisable to seek the advice of the attending physician since it is not always possible to obtain a complete medical history from the patient. Therefore, in the patient without further risk factors, it seems appropriate not to suspend the DOAC, carry out the intervention at the time farthest from the last intake (24 h for OD and 12 h for BID,) and resume the DOAC on the same day once the hemostasis it is stable.

### 2.17. Local Anesthesia

The use of all common local anesthetics is reported in the literature: articaine with vasoconstrictor 1:200,000, and mepivacaine without vasoconstrictor [35,39].

Although the use of epinephrine does not raise concerns about possible systemic effects, its use could cause evaluation errors; in fact, the reduced bleeding due to vasoconstriction could be mistakenly confused with the achievement of adequate hemostasis. Therefore it seems more appropriate, if the surgical parameters are favorable, the use of anesthetic without vasoconstrictor [39].

### 2.18. Improvement of Coagulation

Post-operative bleeding complications after oral surgery occurs significantly more often in patients under continued rivaroxaban therapy (11.5%) than in the control cases without anticoagulation/ antiplatelet medication (0.7%) [20].

According to the EHRA guidelines, since oral surgery is considered low-risk for bleeding due to the possibility of direct hemostasis, time is considered a helping factor in achieving hemostasis; after cessation of treatment, restoration of hemostasis is to be expected within 12–24 h after the last taken dose, given plasma half-life of around 12 h for most DOACs [28].

The half-life of dabigatran is only 12 to 14 h so, given adequate renal function, within 12 h of a dose of dabigatran etexilate (150 mg) at steady state, plasma concentrations are approximately 60 ng/mL (corresponding to an aPTT about 1.5-times baseline) [25,58].

Possible precaution to promote adequate hemostasis are listed in Table 8 [6,20,35,39].

Supportive strategies to control mild bleeding include local wound compression with tranexamic acid gauze; in case of moderate bleeding surgical revision under local anesthesia and accurate suturing and use of tranexamic acid together with gelatin sponges is suggested [39]. Supportive strategies to control severe bleeding include delayed administration of the next dose of dabigatran or discontinuation, maintenance of adequate diuresis, mechanical compression, electrocoagulation, surgical hemostasis, and transfusion of blood products (packed red cells) [35,38,42]. In case of DOACs, the plasma abundance of the DOAC may block newly administered coagulation factors as well. Hence, fresh frozen plasma cannot be considered a reversal strategy. On the other hand, coagulation factor concentrates can be used for reversal [28]. In addition, because of low plasma protein binding, dabigatran is dialyzable. In a study that included patients who had access to emergency medical service following oral surgery, patients receiving DOAC needed more surgery to achieve hemostasis and longer hospitalization but did not require more blood transfusions compared to non-anticoagulated patients [42].

### 2.19. Guideline Proposal for the Management of the DOAC Assuming Patient Undergoing Oral Surgery

**Figure 1:**
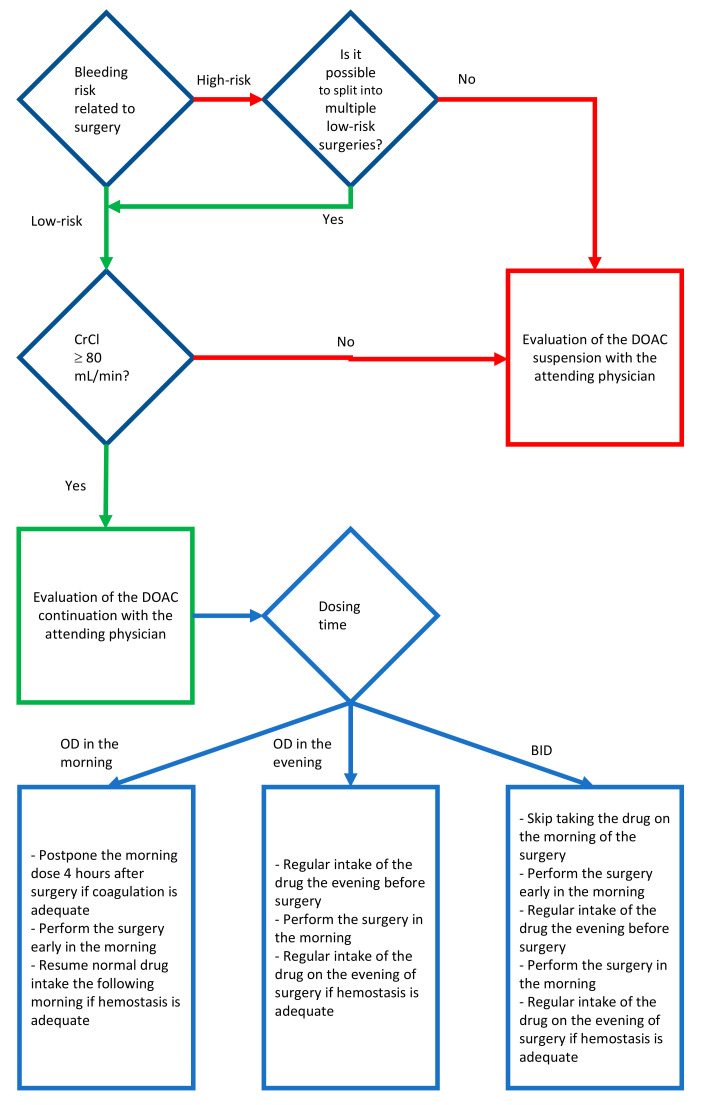
Scheme for the evaluation of the peri-operative maintenance of anticoagulant therapy.

In order to assess the need to discontinue or the possibility of continuing anticoagulant therapy, the first factor to be taken into consideration is the risk of bleeding associated with surgery Figure 1. In the case of surgery with a low risk of bleeding, if the kidney function is adequate, the review of the literature has highlighted the possibility of carrying out the intervention while maintaining anticoagulant therapy. In the case of maintenance of anticoagulant therapy, planning is fundamental. If the patient takes DOAC once a day in the morning, it seems correct to perform surgery early in the morning, before taking DOAC, and postpone taking DOAC until after a stable clot has formed, at least 4 h after surgery. The advantage of this method consists in carrying out the surgery about 24 h after the last intake of anticoagulant. In the event that the patient takes the DOAC once a day in the evening, if possible, gradually move the intake of the drug in the morning and plan the intervention accordingly; if it is not possible to move the intake in the morning, the intake remains unchanged the evening before the intervention, the intervention is carried out in the morning, about 12 h after the last intake, and if in the evening after the surgery the hemostasis is stable the DOAC can be resumed regularly. If the DOAC is taken twice a day, the intake of the evening before the surgery is maintained, the operation is performed in the morning skipping the morning intake of DOAC, and the regular intake of the drug is resumed the evening of the surgery if hemostasis is stable.

If the renal function is reduced, the suspension of the drug in preparation for surgery must be proportional to the reduction in renal function (Table 6). If it is not possible to reduce the invasiveness of the surgery and the risk of associated bleeding, but the patient’s renal function is adequate, it is necessary to stop taking the DOAC 24 h before surgery. If it is not possible to reduce the invasiveness of surgery and the patient’s renal function is reduced, it is necessary to stop taking DOAC before surgery for a period of time proportional to the reduction in renal function (Table 6). After surgery, it is always recommended to resume taking the drug once stable hemostasis is achieved.

Fundamental is the use of adequate aids to hemostasis, such as careful suturing, the use of collagen or oxidized cellulose soaked in tranexamic acid inside the alveolus, the tamponade with gauze soaked in tranexamic acid, cold food and correct hydration in the post-operative period, and use of pain-relieving drugs that do not interfere with coagulation (paracetamol). It is necessary that the oral surgeon is available after the surgery and that the patient has telephone contact.

## 3. Conclusions

The planning of a surgical intervention in a patient taking a DOAC requires knowledge of the pharmacological aspects of the drug and the collection of a scrupulous medical history. These drugs carry a significant burden of adverse and serious effects, and the oral surgeon’s role is to offer patients adequate and safe treatment, promoting health and improvements in their quality of life. In interventions at low bleeding risk, if the patient’s state of health allows it, it is considered correct to maintain the patient’s anticoagulative status. In higher risk cases it may be necessary to replace oral anticoagulant therapy with parenteral therapy. Collaboration with the attending physician is crucial in order to avoid iatrogenic complication.

## Figures and Tables

**Table 1:** Indications for DOACs.

Indications	Dabigatran	Rivaroxaban	Apixaban	Edoxaban
Prevention				
Stroke and systemic embolism in patients with NVAF	Yes	Yes	Yes	Yes
Recurrence of DVT and PE in patients who have been previously treated	Yes		Yes	
DVT and PE in patients who have undergone hip/knee replacement surgery	Yes	Yes	Yes	
Recurrence DVT and/or PE in patients at continued risk for recurrent DVT and/or PE after completion of initial treatment lasting at least 6 months		Yes		
Major cardiovascular events (CV death, MI and stroke) in patients with chronic CAD or PAD		Yes (in combination with aspirin)		
**Treatment**				
DVT and PE in patients who have been treated with a parenteral anticoagulant for 5–10 days	Yes	Yes	Yes	

CAD: coronary artery disease; CV: cardiovascular; DVT: deep venous thrombosis; MI: myocardial infarction; NVAF: non-valvular atrial fibrillation; PAD: peripheral artery disease; PE: pulmonary embolism.

**Table 2:** Summary of the pharmacokinetic profile of DOACs after oral administration.

Drug Feature	Dabigatran	Rivaroxaban	Apixaban	Edoxaban
Prodrug	Yes	No	No	No
Target	Thrombin	FXa	FXa	FXa
Absolute bioavailability	3–7%	66% (Almost 100% with food)	>50%	61.8%
Time to maximum plasma concentration	1.5–4 h	2.5–4 h	2 h	1–3 h
Daily administration	BID	OD	BID	OD
Renal clearance	80–85%	35%	25–27%	35–50%
Plasma protein binding	35%	90%	87%	40–59%
Test	ECT, TT	FXa inhibition assay	FXa inhibition assay, dilute prothrombin time	anti-factor Xa activity

BID: twice a day; ECT: ecarin clotting time; FXa: factor Xa; OD: once daily; TT: thrombin clotting time.

**Table 3:** Potential interactions of oral anticoagulants with drugs used or prescribed in dentistry.

Drug	Via	Dabigatran	Rivaroxaban	Apixaban	Edoxaban
**Antacids** **(H2B; PPI; Al-Mg-hydroxide)**	GI absorption	−12/30% PDC (no clinical effect)	No effect	No effect	No effect
**Itraconazole Ketoconazole Posaconazole Voriconazole**	potent P-gp and BCRP competition; CYP3A4 inhibition	+140/150% PDC **CONTRA-INDICATED**	Up to +160% PDC **CONTRA-INDICATED**	+100% PDC **CONTRA-INDICATED**	+87–95% PDC Reduce DOAC dose
**Fluconazole**	Moderate CYP3A4 inhibition	No data	+42% PDC Consider DOAC dose reduction	No data	No data
**Clarithromycin Erythromycin**	moderate P-gp competition and CYP3A4 inhibition	+15–20% PDC Consider DOAC dose reduction	+30–54% PDC Consider DOAC dose reduction	No data	+90% PDC Reduce DOAC dose
**Rifampicin**	P-gp/ BCRP and CYP3A4 inducer	−66% PDC **CONTRA-INDICATED**	Up to −50% PDC **CONTRA-INDICATED**	−54% PDC **CONTRA-INDICATED**	−35% PDC, but with compensatory Increase of Active Metabolites **AVOID IF POSSIBLE**
**Non-COX-selective NSAIDs (Naproxen** **Ibuprofen, Ketoprofen, Fenoprofen, Flurbiprofen) and salicylates**	P-gp competition and pharmaco-dynamic interaction	GI and peptic ulceration and/or perforation, +60% BR **AVOID IF POSSIBLE**			
**NSAIDs**	Pharmacodynamic Interaction	Increased BR **AVOID IF POSSIBLE**	Increased BR **AVOID IF POSSIBLE**	Increased BR **AVOID IF POSSIBLE**	Increased BR **AVOID IF POSSIBLE**
**Opioid**			Increased BR **AVOID IF POSSIBLE**		

BCRP: breast cancer resistance protein; BR: bleeding risk; NSAID: non-steroidal anti-inflammatory drugs; H2B: H2-blockers; PPI: proton pump inhibitor; GI: gastrointestinal; PDC: plasma drug concentration; P-gp: P-glycoprotein.

**Table 4:** DOACs half-life as a function of creatinine clearance.

Renal Function (C_R_C_L_, mL/min)	Dabigatran	Rivaroxaban	Apixaban	Edoxaban
>80	12–17 h	5–9 h (young) 11–13 h (elderly)	9–12 h	10–14 h
>50 to ≤80	17 h	8.7 h	14.6 h	8.6 h
>30 to ≤50	19 h	9 h	17.6 h	9.4 h
>15 to ≤30	28 h	9.5 h	17.3 h	16.9 h
≤15	No data			

**C_R_C_L_**: creatinine clearance.

**Table 5:** Parameters for indication of continuation of anticoagulation.

Surgical	Metabolic
Extraction of one to three single-rooted teeth Extraction of one multi-rooted teeth Positioning of one to three implants in the anterior region Positioning of one to two implants in the posterior region Periodontal surgery Incision of abscess Flap limited to the attached gingiva and not involving the free gingiva	Good renal function





**Table 6:** Interruption of DOAC therapy before oral surgery.

Renal Function (C_L_C_R_ mL/min)	Dabigatran		Rivaroxaban–Apixaban–Edoxaban	
	Low-Risk Surgery	High-Risk Surgery	Low-Risk Surgery	High-Risk Surgery
≥80	24 h	48 h	24 h	48 h
50–79	36 h	72 h	24 h	48 h
30–49	48 h	96 h	24 h	48 h
15–29	Not indicated	Not indicated	36 h	48 h
<15	No official indication for use			

Adapted from [28,56].

**Table 7:** Frequency and severity of bleeding in previously published clinical trials.

Study	*N* of Procedures	Bleeding (%)	Degree	Dabigatran	Rivaroxaban	Apixaban	Edoxaban
Miranda et al. 2016 [3]	12	0		N.A.	N.A.	N.A.	N.A.
Clemm et al. 2016 [35]	16	0		0/6	0/8	0/2	
Hanken et al. 2016 [20]	52	11.5	Two local compressions, four reoperations		6/52		
Mauprivez et al. 2016 [29]	32	17.8	85.7% local compression, 14.3% reoperation	2/9	1/21	2/2	
Morimoto et al. 2016 [38]	19	31.6	five local compression, one reoperation	0/4	3/9	3/6	
Yagyuu et al. 2017 [37]	41	9.7	Minor bleeding	N.A.	N.A.	N.A.	N.A.
Lababidi et al. 2018 [6]	38	10.5	Minor bleeding	N.A.	N.A.	N.A.	N.A.
Yoshikawa et al. 2019 [31]	128	3.1	50% local compression, 50% reoperation	1/37	0/34	2/39	1/18
Berton et al. 2019 [36]	65	18.5	Local compression	1/11	3/28	1/22	0/4

**Table 8:** Precautions to improve coagulation.

Accurate Suturing Technique
Irrigation of the alveoli by antifibrinolytic agents such us tranexamic acid together with gelatin sponges
Collagen and resorbable oxycellulose applied into the extraction socket
Acrylic splint for wound protection
Application of fibrin glue and secondary sutures
Accurate alveolar bone cleaning to remove bleeding granulation tissue
Post-operative wound compression with gauze soaked in tranexamic acid
Ice pack
Mouth rinses with a 10 mL of 5% tranexamic acid aqueous solution for 2 min, repeated four times daily for seven days
Cold food and avoid mouthwashes
Avoid vertical releasing incisions if possible
